# Animal Models of Aortic Aneurysm and Dissection: A Comparative Guide for Mechanism, Therapeutic Testing, and Translational Readouts

**DOI:** 10.3390/biomedicines14010170

**Published:** 2026-01-13

**Authors:** Shayan Mohammadmoradi, Sidney W. Whiteheart

**Affiliations:** 1 Saha Cardiovascular Research Center, University of Kentucky, Lexington, KY 40536, USA; 2 Department of Molecular and Cellular Biochemistry, University of Kentucky, Lexington, KY 40536, USA

**Keywords:** abdominal aortic aneurysm, thoracic aortic aneurysm, aortic dissection, angiotensin II, elastase, BAPN, mineralocorticoid receptor, fludrocortisone, TGF-β, platelets

## Abstract

Aortic aneurysms and dissections are devastating vascular diseases with high mortality, yet no pharmacological therapy has proven effective in halting growth or preventing rupture. Surgical and endovascular repair remain the only treatment options for advanced disease. Animal models have been indispensable in defining mechanisms and testing candidate therapies, but the diversity of protocols, strain-dependent variability, and heterogeneous endpoints complicate interpretation and translation. This review provides an update focused on how to match models to specific research questions. We critically compare commonly used abdominal aortic aneurysm (AAA) models (angiotensin II ± hyperlipidemia, elastase, calcium chloride, β-aminopropionitrile BAPN hybrids, and mineralocorticoid agonist/fludrocortisone models) with thoracic aortopathy and dissection models (BAPN alone or with AngII, genetic models including Marfan and smooth muscle contractile mutations, and AngII + TGF-β blockade). We highlight practical considerations on segment specificity, rupture incidence, lipid dependence, comorbidities, and outcome measurement, with emphasis on rigor and reporting standards. A translational thread on platelet–intraluminal thrombus biology, including the emerging biomarker and therapeutic targets such as glycoprotein VI (GPVI), is integrated across models. We offer a decision grid and rigor checklist to harmonize model use, enhance reproducibility, and accelerate translation.

## 1. Introduction

Aortic aneurysms and dissections (AAD) remain among the most lethal vascular diseases worldwide [1,2,3]. Abdominal aortic aneurysm (AAA) affects up to 5% of men over age 65, and rupture carries a mortality rate exceeding 70% despite advances in surgical and endovascular repair [2,3]. Thoracic aortic aneurysms (TAA) are less common but equally devastating, often presenting as acute dissection in younger individuals with heritable thoracic aortic disease (HTAD) [4]. Together, these conditions cause tens of thousands of deaths annually and constitute a major global health burden [1,5]. Unlike atherosclerosis or hypertension, where decades of research have delivered pharmacological therapies, no medical treatment has been proven to slow aneurysm growth or prevent rupture. Surgery remains the only effective option once aortic dimensions reach threshold criteria [2,4]. This persistent gap defines aneurysm and dissection as “therapeutically orphan” diseases.

The pathophysiology of AAA and TAA reflects both shared mechanisms and distinct biology. Both involve extracellular matrix (ECM) degradation, smooth muscle cell dysfunction, and inflammatory remodeling [4,5,6,7,8]. However, AAA is strongly linked to acquired risk factors, age, smoking, hypertension, hyperlipidemia, and typically occurs infrarenally, often with intraluminal thrombi [7,9,10]. By contrast, TAA frequently involves the ascending aorta, often arises in younger patients, and is commonly driven by genetic mutations affecting fibrillin-1 (*FBN1*), TGF-β receptors (*TGFBR1/2*), or smooth muscle contractile proteins (ACTA2, MYH11) [4,5,11]. These differences highlight why one-size-fits-all models cannot capture the complexity of human aortopathies. Animal models have therefore been indispensable in dissecting pathophysiology and enabling therapeutic testing [12].

The field of experimental aortic disease has evolved through distinct eras. In the 1990s, the angiotensin II (AngII) infusion model in hyperlipidemic mice emerged as a workhorse for rapid AAA induction and rupture studies [13]. Around the same time, elastase perfusion and calcium chloride injury were developed as the first reproducible infrarenal AAA models, mimicking the human anatomical location but producing relatively low rupture incidence [14]. Subsequently, exposure to β-aminopropionitrile (BAPN), a lysyl oxidase inhibitor, was recognized as a potent inducer of thoracic aortic disease, revealing striking regional differences between the ascending and descending aorta [15]. More recently, mineralocorticoid agonist models using deoxycorticosterone acetate (DOCA), aldosterone, or fludrocortisone demonstrated that aneurysms can develop through blood pressure-independent, endocrine-driven pathways [16,17]. Finally, the advent of CRISPR/Cas9 genome editing has enabled precise knock-in of human HTAD mutations, ushering in a new era of mutation-specific and precision-guided models [18]. Together, these models illustrate the creativity of the field but also highlight the challenge: each reproduces only a segment of the human disease spectrum.

Despite decades of progress, the field faces major challenges. Outcomes differ dramatically by model, substrain, sex, and age, limiting reproducibility across laboratories. Therapeutic results are often model-dependent: drugs protective in one model may fail or even worsen outcomes in another [8,19,20,21,22]. Translational endpoints also remain underdeveloped: most studies rely on diameter, while human disease is often driven by rupture and influenced by intraluminal thrombosis [2]. Prior reviews have cataloged these models in isolation [4,5,6,7,8,23,24,25,26,27,28], but several challenges remain: (i) the need to match models to specific scientific questions; (ii) the importance of rigor and reproducibility standards; and (iii) the lack of translational threads connecting animal model biology to human disease features such as an intraluminal thrombus (ILT) and platelet activity. By integrating these perspectives, this review aims to provide a roadmap for investigators to navigate the expanding landscape of aortic aneurysm models and move preclinical work closer to effective clinical therapies.

## 2. Abdominal Aortic Aneurysm Models

AAA remains the most extensively studied form of aortic disease in preclinical research [7]. Experimental models have been pivotal in uncovering mechanisms of extracellular matrix degradation, vascular inflammation, and rupture biology, as well as in providing platforms for therapeutic testing [26]. Yet, each model reflects only a subset of the human condition. A recent review has provided detailed accounts of rodent AAA models [6]; here, we offer a concise summary of the major systems, highlighting their induction methods, pathophysiological features, strengths, and limitations in relation to human disease.

### 2.1. Angiotensin II Infusion

AngII infusion in hyperlipidemic backgrounds (ApoE^−/−^, LDLR^−/−^, or AAV-PCSK9 in WT mice) produces suprarenal AAAs within 28 days [24,26,27,29,30,31,32,33]. In addition to abdominal pathology, AngII also induces thoracic aortic aneurysms and dissections, particularly in the ascending and arch regions of hyperlipidemic mice. Thoracic outcomes are less frequent and more variable, depending on strain and lipid status [4]. This model is highly reproducible, rupture-prone, and widely used to study immune, metabolic, and platelet mechanisms [6,7]. It is ideal for rapid drug testing and survival endpoints but does not replicate the infrarenal location typical of human AAA. Outcomes are strongly influenced by substrain (C57BL/6J vs. 6N), age, sex, and lipid status [34].

### 2.2. Elastase

Intraluminal or periadventitial elastase application induces infrarenal aortic dilation with prominent elastin fragmentation [35]. This model closely resembles the anatomic site of human AAA and is suitable for matrix and biomechanics studies. However, rupture is rare unless combined with BAPN [36]. Surgical skill and mouse substrain strongly affect reproducibility.

### 2.3. Calcium Chloride

Periadventitial CaCl_2_ application induces consistent infrarenal dilation and ECM remodeling [37,38]. It is technically simpler than elastase and produces stable aneurysms but rarely causes rupture. It is best suited for ECM biology and non-rupture growth studies.

### 2.4. Elastase + BAPN Hybrids

Combining elastase injury with BAPN administration generates rupture-prone infrarenal AAAs with ILT, closely mimicking advanced human pathology [39]. This is highly useful for rupture biology and ILT studies but requires young animals and careful dosing to avoid excessive mortality.

### 2.5. Mineralocorticoid Receptor

Mineralocorticoid receptor (MR) agonists such as deoxycorticosterone acetate (DOCA) or aldosterone, given with high salt, induce both abdominal and thoracic aneurysms in C57BL/6 mice [16]. Lesions form in the suprarenal and descending thoracic aorta, with significant rupture rates, and show hallmarks of human disease, including elastin degradation, smooth muscle loss, oxidative stress, and inflammation. Notably, aneurysm formation occurs independently of blood pressure and is prevented by MR antagonists, but not by ACE inhibitors or ARBs, highlighting endocrine-specific mechanisms. This model is valuable for exploring MR-driven vascular remodeling and testing MR blockade; however, its reliance on high-salt intake and predominance of suprarenal lesions limit its ability to fully recapitulate human infrarenal AAA.

### 2.6. Large-Animal Models

Large-animal systems provide an essential bridge for translational device testing and hemodynamic studies. Rabbit AAA models, created by intraluminal elastase or elastase–collagenase perfusion, allow reproducible infrarenal dilation suitable for wall stress and flow analyses, though rabbits are less suitable for full-scale device deployment [40]. Porcine models more closely approximate human aortic dimensions and mechanics, supporting realistic deployment of stent grafts and flow measurements [41]. Both thoracic and abdominal porcine aneurysm models have been described, including surgically created saccular dilations and pharmacologic induction [41,42]. These models are best suited for device and biomechanical testing rather than mechanistic studies, with AAA being more common in rabbits and both AAA and TAA platforms available in swine.

## 3. Thoracic Aortic Aneurysm and Dissection Models

TAA and dissection models complement AAA systems by capturing the unique biology of the thoracic aorta, where genetic mutations, developmental factors, and hemodynamic stress are dominant drivers. Unlike AAA, which is largely associated with acquired risk factors, TAA often arises from heritable syndromes or progressive medial degeneration. The recent American Heart Association (AHA) guidelines provide detailed recommendations for designing and reporting preclinical thoracic aortopathy studies, underscoring the need for rigor and standardization in this field [4]. In this section, we summarize the principal thoracic models, including pharmacological and genetic HTAD strains, and highlight how each has advanced our understanding of thoracic aortopathy and its translational challenges.

### 3.1. BAPN

BAPN, an irreversible lysyl oxidase inhibitor, disrupts elastin and collagen crosslinking, weakening the aortic wall. In young mice, it induces ascending aortic dilation and descending dissections with a high incidence of rupture, reflecting region-specific vulnerabilities linked to smooth muscle cell origin and matrix architecture [15]. Disease is age-dependent, appearing mainly in immature mice, and varies by strain. Most therapeutic interventions, including cilostazol and sildenafil, have been ineffective, highlighting the model’s utility for dissecting elastin biology and dissection mechanisms rather than drug discovery [19]. Its strengths are reproducibility and mechanistic clarity, while limitations include restriction to young animals, absence of abdominal disease, and poor translational response to candidate therapies.

### 3.2. BAPN + AngII

Combining BAPN with AngII infusion markedly amplifies disease severity, producing rapid thoracic dilation, intramural hematomas, and high rupture rates [8,43]. Typically applied in very young C57BL/6 mice, BAPN is administered via drinking water, diet, or injection, followed by AngII infusion for days to weeks. Concurrent treatment accelerates dissection onset, while staged infusion increases overall incidence. Outcomes are sex-dependent, with males showing greater expansion and medial destruction [4]. This aggressive model is valuable for studying acute dissection biology and testing short-term stabilization strategies, but its high incidence of early mortality, reliance on immature animals, and narrow therapeutic window limit its use for chronic or mechanistic studies.

### 3.3. AngII + TGF-β Blockade

Combining AngII infusion with TGF-β neutralization or receptor deletion produces aggressive thoracic dissections with rapid rupture [4,21]. This model consistently yields widespread medial degeneration, intramural hematomas, and false lumen formation, underscoring the critical role of TGF-β signaling in maintaining thoracic aortic integrity. The severity of disease far exceeds that seen with AngII or TGF-β perturbation alone, highlighting a synergistic interaction between hemodynamic stress and impaired repair signaling. This model is valuable as one of the most stringent preclinical stress tests for candidate therapies, capable of exposing adverse or paradoxical effects that may not appear in less aggressive systems. However, its rapid lethality, technical difficulty, and limited window for intervention constrain its utility for studying chronic progression. As such, it is best suited for mechanistic studies of TGF-β biology and for short-term therapeutic testing under conditions that closely mimic catastrophic human dissection.

### 3.4. Fludrocortisone

Fludrocortisone, a synthetic mineralocorticoid, induces thoracic aortic dilation, wall thickening, and occasional hemorrhage in C57BL/6J and ApoE^−^/^−^ mice, largely independent of hypercholesterolemia or blood pressure [17]. This model highlights endocrine, mineralocorticoid receptor-driven pathways and complements DOCA and aldosterone systems. Its strengths lie in revealing non-traditional mechanisms of aortic remodeling with clinical relevance, though its limitations include predominant thoracic involvement, rare rupture, and limited relevance to human aortopathies [17].

### 3.5. Genetic HTAD Models

Genetic models of heritable thoracic aortic disease (HTAD) remain among the most powerful tools for dissecting mechanisms of thoracic aortopathy. Classic examples include *Fbn1* mutations that model Marfan syndrome, producing root and ascending dilation with progressive medial degeneration [8,44]. *Tgfbr2* mutations, central to Loeys–Dietz syndrome [22,28], and smooth muscle contractile gene variants such as MYH11 further underscore the role of impaired contractility and dysregulated TGF-β signaling in ascending aortic disease [45]. Beyond these canonical genes, smooth muscle-specific deletion of *Lrp1* (low-density lipoprotein receptor-related protein 1) has emerged as a robust model of thoracic aneurysm [28,46,47]. *Lrp1* deficiency destabilizes extracellular matrix homeostasis, leading to enhanced susceptibility to AngII-induced aneurysm and dissection [47].

These models have been invaluable for confirming causative mutations, testing therapies (e.g., losartan in *Fbn1* mutants), and highlighting pathways such as TGF-β and SMC contractility. Their limitations include variable penetrance, lack of consistent rupture endpoints, and divergence from comorbidity-rich human disease. Recent AHA/ATVB guidelines stress rigorous control of sex, strain, and age, and transparent reporting [4]. Thus, while indispensable, genetic HTAD models must be interpreted with both their strengths, faithful recapitulation of human mutations, and their limits in translational predictiveness.

## 4. Principles for Designing, Interpreting, and Reporting Aneurysm Studies

Preclinical aneurysm research is as much about how models are applied as models chosen. Here, we outline the principles that should guide experimental design, from accounting for biological variability and comorbidities, to ensuring rigorous imaging and biomechanical endpoints, to recognizing the importance of publishing negative results:

### 4.1. Cross-Model Biology: What’s Conserved and What’s Context-Dependent

Across AAA and TAA/dissection models, several mechanistic threads recur, yet their relative weights differ with induction method, vascular segment, and disease stage. Innate immunity is a common denominator: neutrophil recruitment, NET formation, and macrophage remodeling (M1/M2 skewing) are consistently observed in AngII AAA and elastase-based infrarenal models [6,35,38,39,48,49], while BAPN-driven thoracic disease often shows a more abrupt inflammatory signature aligned with medial failure and intramural hematoma. Smooth muscle cell (SMC) phenotypic modulation, from contractile to synthetic or osteochondrogenic states, is evident across models [15,19,36,43,50], but is particularly prominent in genetic TAA (e.g., *Fbn1*, *Tgfbr2*) where cytoskeletal signaling and mechano-transduction defects are primary drivers [8,20,51]. ECM homeostasis—elastin fragmentation, collagen remodeling, and crosslink integrity unifies all models; however, the route to failure differs: enzymatic injury dominates elastase/CaCl_2_ AAA [10,36,37,38,52]; impaired crosslinking precipitates BAPN dissections [15,19,50]; and coupled immunometabolic stress intensifies AngII AAA [7,10]. This diversity argues against a single “master pathway” and supports a modular view of aneurysm biology: immune–SMC–ECM modules engage in different proportions depending on the model and question at hand. Practically, this means therapies targeting broadly conserved axes (e.g., protease suppression, oxidative stress moderation) may generalize more readily than those aimed at model-specific triggers (e.g., LOX inhibition context), and that multimodel validation is essential before clinical translation. In addition to immune activation and structural degeneration, accumulating evidence identifies vascular SMC metabolic reprogramming and epigenetic regulation as central, integrative drivers of chronic aneurysm progression [53]. Multi-omics analyses of human AAA tissue and complementary murine models demonstrate a shift toward enhanced glycolysis, impaired mitochondrial oxidative phosphorylation, and altered redox balance within SMCs, accompanied by durable changes in chromatin accessibility and transcriptional state [54]. These metabolic–epigenetic programs stabilize maladaptive SMC phenotypes, promote extracellular matrix turnover, and sustain inflammatory signaling over prolonged periods, providing a mechanistic substrate for slow aneurysm growth independent of acute injury [53]. Importantly, this regulatory layer offers insight into why high-stringency or rupture-prone models, while invaluable for studying wall failure, may incompletely recapitulate the gradual, phase-dependent remodeling observed in human disease [55]. Incorporating metabolic and epigenetic context into the immune–SMC–ECM framework emphasizes that aneurysm biology reflects not only inflammatory burden or matrix degradation, but long-lived cellular reprogramming, with direct implications for model selection, endpoint definition, and identification of stage-specific therapeutic targets.

### 4.2. Biological Sources of Variability and How to Control Them

Sex, age, and genetic background are not nuisance covariates; they are determinants of phenotype. Male bias in AAA incidence is mirrored in AngII and elastase models [6,25,27,29,38,56,57], yet female cohorts can reveal distinct inflammatory and matrix signatures and different drug responses. Age modifies susceptibility and rupture; older mice exhibit higher aneurysm penetrance in mineralocorticoid/fludrocortisone models and greater mortality under high-stringency BAPN protocols [15,58,59]. Equally important are substrain and vendor effects, the C57BL/6J vs. 6N dichotomy [34] and even supplier-specific microbiome differences can shift AngII AAA incidence, lesion distribution, and survival [60].

Constructively, we recommend: (i) a priori inclusion of sex as a factor with powered subgroup analyses; (ii) explicit reporting of substrain and vendor; (iii) minimizing cross-vendor mixing mid-study; and (iv) pretesting age bands for high-stringency models (BAPN, elastase + BAPN) to avoid ceiling mortality. Treating these variables as design features rather than afterthoughts will markedly improve reproducibility across laboratories.

Across models, sex consistently influences aneurysm incidence, progression, and rupture. In AngII-driven AAA, males exhibit higher penetrance and rupture risk, whereas females are relatively protected, in part through sex hormone-dependent mechanisms [6]. In infrarenal elastase and calcium chloride models, sex differences in dilation are less pronounced but emerge at the level of inflammation and matrix remodeling. In thoracic models, including BAPN-based systems, males again show greater susceptibility to dissection and rupture [19]. These patterns underscore the importance of treating sex as a biological variable at the design stage rather than as a post hoc covariate.

### 4.3. Comorbidities and Environmental Modifiers

Human AAA rides on a burden of lipids, smoking, and hypertension, whereas TAA often rides on genetics and hemodynamics [1]. Models can and should reflect this. Hyperlipidemia (ApoE^−/−^, LDLR^−/−^, or AAV-PCSK9) strengthens AngII AAA and helps interrogate immunometabolism; saline or high-salt loads tune endocrine models; and BAPN’s sensitivity to age and hydration must be respected [6,7,33]. Two often-ignored modifiers merit attention. First, diabetes: epidemiologically linked to lower AAA growth, yet rodent hyperglycemia can variably increase ECM turnover, clarify whether the question is about initiation versus progression, and report glycemic control longitudinally [58,61,62,63,64]. Second, microbiome differences between facilities/vendors alter immune tone and can shift aneurysm penetrance [60,65,66]; cohousing or bedding exchange are pragmatic ways to reduce site effects. It is important to purposefully choose comorbidities and document them as first-class variables.

### 4.4. Imaging, Measurement, and Validation: From “Diameter” to “Decision-Grade Data”

Diameter alone is a blunt instrument. At minimum, report serial ultrasound growth rates (mm/week) with probe frequency, imaging planes, heart rate, and temperature, and ensure blinded acquisition and analysis [4]. Serial ultrasound is generally sufficient for longitudinal growth assessment in stable models; however, in rupture-prone protocols, end-stage analyses, or inter-laboratory comparisons, in situ perfusion-fixed measurements or OCT-based validation are strongly recommended to minimize measurement artifacts. In rupture-prone protocols, prespecify humane endpoints and use Kaplan–Meier survival with necropsy adjudication (aneurysm rupture vs. other causes). Because post-mortem vasoconstriction and loss of turgor can artifactually shrink diameters, in situ validation should use perfusion or OCT-based approaches that preserve lumen geometry [67]. For inter-lab comparability, include a calibration phantom or repeated measures on a WT control cohort to estimate measurement error. Finally, align outcomes with the biological question: when studying biomechanical failure, couple imaging with ex vivo tensile testing (failure stress/strain, elastic modulus, burst pressure); when testing anti-inflammatory agents, include standardized histology panels (elastin fragmentation, macrophage density, ILT composition) with blinded scoring. These steps convert imaging from a figure-friendly snapshot to decision-grade data suitable for model selection and therapy gating. Endpoint selection should be guided by the scientific question. Growth rate is most appropriate for studies focused on chronic aneurysm progression or therapeutic slowing of disease, whereas survival and rupture endpoints are essential for interrogating wall failure and stabilization mechanisms. Biomechanical testing is best suited for questions centered on material integrity and extracellular matrix strength, while analysis of intraluminal thrombus composition and platelet activation is most informative for studies of inflammatory–thrombotic crosstalk. Reliance on diameter alone risks obscuring these distinctions.

### 4.5. Translational Platelet and Intraluminal Thrombus Biology

ILT is present in most large human AAAs, shaping wall hypoxia, inflammation, and protease activity [9,68,69]. Platelets drive ILT formation and have phase-specific roles. Early depletion or inhibition worsen rupture, suggesting a hemostatic stabilizing effect [24,70]. In established disease, platelet activation promotes progression; targeting GPVI reduces AAA growth and mortality in mice [71]. Soluble GPVI (sGPVI) levels predict AAA growth in patients, outperforming D-dimer as a biomarker [70,71,72]. Incorporating platelet readouts aligns murine findings with human monitoring and therapeutic targeting. Similar translational tensions have been observed in other pathways, including TGF-β signaling and antiplatelet strategies, where interventions protective in select animal models worsened outcomes in others or conflicted with human observational data [6,9,20,21]. These examples reinforce the need for phase-specific interpretation and multi-model validation when translating preclinical findings.

### 4.6. Publishing the “No”

Overemphasis on positive diameter outcomes has contributed to fragile science in the field. Null or negative findings—such as the lack of benefit from cilostazol or sildenafil in BAPN-induced TAA [19], or the increased rupture risk with early platelet inhibition, [70] serve as critical safeguards against misleading conclusions. To strengthen the evidence base, one should incorporate explicit futility thresholds (e.g., stopping rules when conditional power falls below 20%) and negative datasets, including imaging and histology, into public repositories or manuscript supplements to ensure transparency and cumulative learning.

### 4.7. Practical Dimensions: Time, Cost, and Operator Dependence

Beyond biology, investigators must also weigh logistical factors such as time-to-endpoint, throughput, cost, and operator expertise. As a general guide: AngII-induced AAA is rapid, high-throughput, and relatively low-skill; elastase AAA requires moderate time and technical proficiency, with low rupture rates; elastase + BAPN increases rupture incidence but demands greater surgical skill and monitoring; BAPN thoracic models develop quickly with high rupture rates but require careful oversight; and MR/fludrocortisone models proceed on a moderate timeline with added complexity from dietary and endocrine variables. Encoding these considerations into the decision framework (Table 1) enhances its utility for experimental planning and grant preparation.

## 5. Decision Grid for Model Selection

Given the diversity of available models, a critical challenge for investigators is selecting the system most appropriate for their scientific question. No single model captures the full complexity of human abdominal or thoracic aortopathies, and reliance on a single approach can lead to misleading or non-translatable results. To address this, we provide a decision grid (Table 1) that aligns specific research questions with the models most suitable to answer them. For example, studies focused on rupture biology are best served by rupture-prone combinations such as elastase plus BAPN in the infrarenal aorta, or BAPN ± AngII in the thoracic aorta. Conversely, if the aim is to study progressive but stable aneurysm growth, models such as AngII infusion in hyperlipidemic mice, elastase injury, or calcium chloride application provide reproducible dilation without high rupture rates. For genetic mechanisms of heritable thoracic aortic disease, models carrying *Fbn1*, *Tgfbr2*, or *Myh11* mutations are indispensable, whereas endocrine pathways can be probed in models driven by mineralocorticoid agonists or fludrocortisone. Finally, when translational device testing or hemodynamic studies are the priority, large-animal models (rabbit or porcine) are the most appropriate bridge. The grid emphasizes a central theme of this review: the “best” model is not universal, but conditional on the research objective. By explicitly linking biological questions with experimental systems, investigators can minimize model–question mismatch, increase reproducibility, and improve the translational value of their findings.

To aid practical interpretation, commonly used models also differ in approximate timelines and severity. For example, AngII infusion in hyperlipidemic male mice typically produces detectable abdominal aneurysms within 1–2 weeks, with rupture occurring in a substantial subset over 3–4 weeks [39]. Elastase- or calcium chloride-based infrarenal models generally produce stable dilation over similar timeframes but rarely rupture unless combined with lysyl oxidase inhibition [19]. In contrast, BAPN-based thoracic models and BAPN–AngII combinations induce rapid disease with high early mortality, often within days [15]. These values are provided as qualitative guides rather than fixed benchmarks, as outcomes vary with sex, strain, age, and protocol details.

## 6. Rigor and Reporting Checklist

A persistent challenge in preclinical aneurysm research is the lack of standardized reporting and methodological rigor. Small differences in substrain, diet, age, or sex can markedly influence outcomes, yet these variables are often underreported. In addition, inconsistent imaging protocols, endpoint definitions, and statistical approaches undermine reproducibility across laboratories. Recent ATVB Council consensus statements have highlighted the need for transparent, harmonized standards in thoracic aortopathy studies [4], but comparable guidelines for AAA research remain less developed and urgently needed [6].

**Figure 1:**
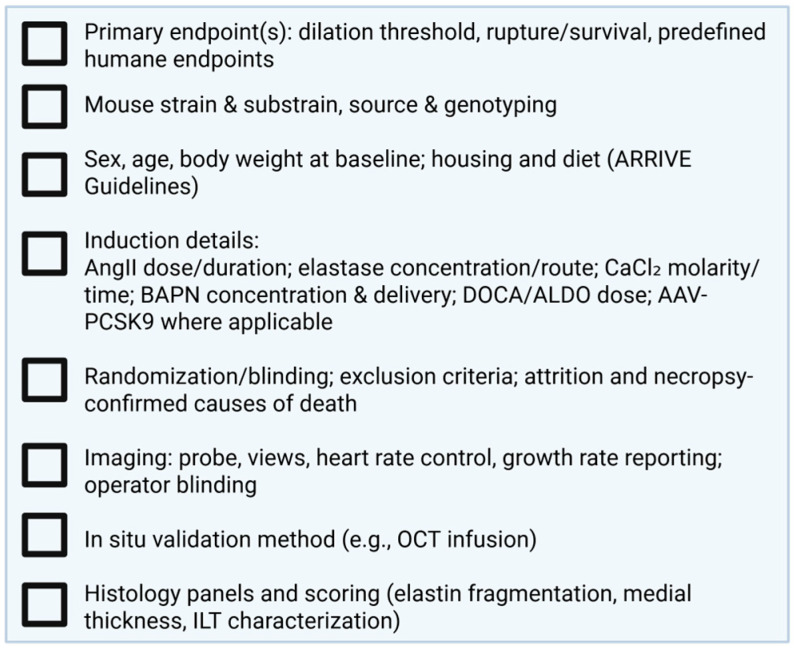
Rigor and Reproducibility Checklist.

To address these challenges, we propose a rigor and reporting checklist (Figure 1) that consolidates key design and reporting items. Essential variables include documentation of mouse substrain, sex, age, body weight, induction protocol details (dose, route, duration), and comorbidity context (diet, lipid status, salt intake). Critical methodological elements—randomization, blinding, power analysis, and prespecified exclusion criteria, should be explicitly reported, as many journals now require adherence to ARRIVE guidelines for animal research. Yet, compliance within the aneurysm field remains inconsistent with key details often missing from AAA and TAA studies. Greater alignment with ARRIVE principles would improve reproducibility, facilitate cross-study comparisons, and strengthen confidence in translational findings. For outcomes, the checklist emphasizes growth rate quantification by ultrasound, validation of aortic diameters using in situ or OCT-based methods, standardized histological scoring for matrix integrity and intraluminal thrombus, and clear criteria for rupture and survival endpoints. The purpose of this framework is not prescriptive uniformity but consistent transparency, enabling meaningful comparisons across studies and rigorous evaluation by readers and reviewers. Adoption of such standards will align preclinical aneurysm research with broader efforts in biomedical science, where reproducibility and transparency are now central expectations. In particular, recent NIH policies on rigor and reproducibility, including requirements for authentication of biological resources, sex as a biological variable, and transparent reporting of experimental design, underscore the urgency of applying similar standards in cardiovascular and aneurysm research. Integrating these principles will help elevate preclinical work to the level of reliability necessary for successful translation to clinical investigation.

## 7. Therapeutic Testing: Lessons Learned

Therapeutic testing across aneurysm models has yielded both encouraging signals and cautionary failures. Mineralocorticoid receptor antagonists consistently prevent aneurysm formation in DOCA-, aldosterone-, and fludrocortisone-driven models, establishing endocrine pathways as actionable therapeutic targets [16,17]. Other promising strategies include the inhibition of mPGES-1 [73] and blockade of platelet glycoprotein VI (GPVI) [71], both of which reduce AAA growth and rupture in AngII and elastase/BAPN models. At the same time, many interventions that appeared protective in one system have failed in others. Cilostazol (PDE3 inhibitor) and sildenafil (PDE5 inhibitor), for example, showed no benefit in BAPN-induced TAA [19] despite earlier success in AAA models [74,75]. Similarly, global platelet inhibition early in disease unexpectedly increased rupture risk, underscoring that platelet roles are phase-dependent [68]. Perhaps most striking, neutralization of TGF-β signaling, once considered a rational therapeutic approach, dramatically accelerated rupture in AngII-infused mice [20,21,22], overturning long-standing assumptions and highlighting the protective role of TGF-β in thoracic aortic integrity [4].

Collectively, these experiences highlight three enduring lessons for the field: (i) therapeutic efficacy is highly model-specific, and results from a single system should not be overgeneralized; (ii) endpoints must move beyond diameter alone to reflect clinically meaningful outcomes such as growth rate, rupture, and survival; and (iii) timing of intervention is critical, as drugs tested at disease initiation may behave very differently in established aneurysms.

Together, these principles argue for multi-model validation, rigorous endpoint selection, and phase-specific study designs to strengthen the translational value of preclinical testing. Importantly, these lessons also provide a roadmap: by learning from past failures and refining experimental design, the field is now better positioned to identify therapies with genuine translational promise.

## 8. Discussion

Animal models of aortic aneurysm and dissection have provided extraordinary mechanistic insights, yet their diversity and limitations underscore a persistent translational gap. The AngII infusion model remains the dominant system for AAA research due to its speed, reproducibility, and rupture incidence, but it predominantly produces suprarenal lesions in hyperlipidemic mice, which differ from the infrarenal phenotype most common in humans [6,7,25,29]. Elastase and calcium chloride injury models reproduce infrarenal dilation and matrix degradation but rarely rupture unless combined with BAPN, limiting their use for rupture biology [24,36,38,76]. BAPN itself induces heterogeneous thoracic phenotypes, with dilation in the ascending aorta and dissections or rupture in the descending aorta [15,19]. These region-specific differences emphasize that no single model captures the full clinical spectrum. Thoracic models further highlight paradoxes in biology. AngII combined with TGF-β neutralization or receptor deletion accelerates rupture, demonstrating that TGF-β signaling, once considered pathogenic, also has protective roles [4,21,25]. Genetic HTAD models (*Fbn1*, *Tgfbr2*, *Myh11*) are invaluable for understanding heritable disease, but their penetrance varies by substrain, age, and husbandry, complicating comparisons across laboratories [8,45,77,78].

**Figure 2:**
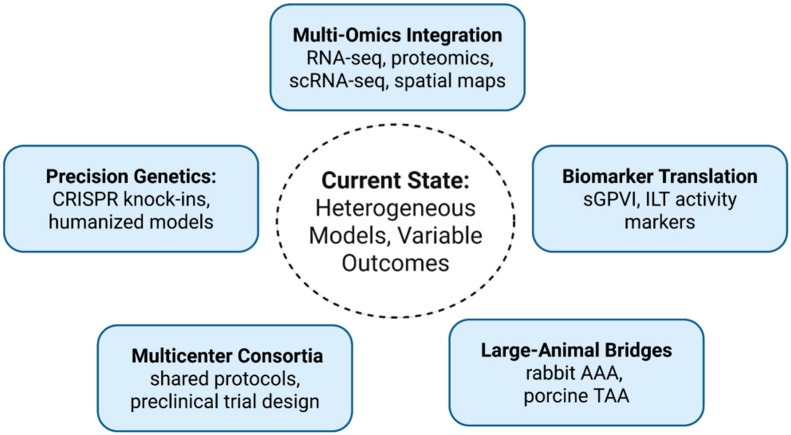
Future Roadmap for Aneurysm Research.

Together, these experiences reinforce the principle that model choice must be guided by the research question, not by habit. Therapeutic outcomes are highly model- and phase-specific, and overgeneralization has led to repeated translational failures. Therefore, the field must move from descriptive use of models toward strategic integration. This includes developing decision frameworks to align models with distinct questions (growth vs. rupture vs. dissection), enforcing rigor checklists to improve reproducibility across laboratories, requiring multimodel validation before advancing therapeutic claims, and embedding translational biomarkers (e.g., soluble GPVI) to align murine endpoints with clinical monitoring. Only through such integration can mechanistic discoveries be converted into therapies that alter patient outcomes. Looking ahead, several priorities define the roadmap for preclinical aortic research (Figure 2).

### 8.1. Precision Genetics and Humanized Models

Advances in CRISPR/Cas9 now allow knock-in of human disease mutations (*FBN1*, *TGFBR2*, *ACTA2*, *MYH11*), enabling precise modeling of HTAD variants. For example, ACTA2 R179H knock-in mice display smooth muscle dysfunction consistent with human thoracic aortopathy [18]. Humanization of immune and platelet pathways offers additional opportunities to model intraluminal thrombus biology with greater translational fidelity. Although costs and husbandry are challenges, these systems will become indispensable for mutation-specific testing.

### 8.2. Multi-Omics Integration

Transcriptomic, proteomic, and spatial analyses of aortic tissue from AngII, elastase, BAPN, and genetic models are increasingly available [11,15,46,68,79,80]. Integrating these datasets across models and species will distinguish conserved mechanisms from model-specific artifacts, generating network-level maps of aneurysm biology. Falling sequencing cost increases feasibility, but collaborative bioinformatics pipelines will be essential to realize this potential.

### 8.3. Biomarker Translation

Soluble GPVI (sGPVI), a platelet-specific biomarker, predicts AAA growth and outperforms D-dimer in patients [71]. Its detection in both mouse and human aneurysm tissue underscores its value as a translational bridge. Future studies should build on this example by integrating biomarkers such as sGPVI with imaging and additional circulating markers to develop risk stratification panels that can guide patient monitoring and clinical trial enrollment.

### 8.4. Multicenter Consortia and Standardization

Variability in substrain, diet, and experimental technique remains a major obstacle [34]. A multicenter preclinical consortium, supported by funding agencies and professional societies, could harmonize protocols, enable blinded imaging repositories, and improve reproducibility. This approach aligns with broader NIH policies on rigor and reproducibility and is a realistic step for the coming decade.

### 8.5. Phase-Specific Therapeutic Strategies

Most patients present with established aneurysms, yet many preclinical studies test therapies only at initiation. Evidence shows that timing is critical: platelet inhibition, TGF-β blockade, and PDE inhibitors have all shown phase-dependent effects [9,70,81,82]. Designing studies that reflect clinical reality, treating established disease, is essential for translational success.

Taken together, these considerations argue for a forward-looking experimental paradigm in which animal models are embedded within a broader, integrative translational framework. While in vivo models remain indispensable for interrogating hemodynamics, rupture biology, and system-level interactions, their limitations in capturing disease chronicity and human heterogeneity highlight the need for complementary human-relevant platforms. Emerging approaches, including patient-derived vascular organoids and microfluidic vascular-on-chip systems, provide controlled environments to interrogate human smooth muscle cell metabolism, epigenetic state, and matrix remodeling under defined biomechanical and inflammatory conditions that are difficult to isolate in vivo [83,84,85]. Importantly, these systems are not intended to replace animal models, but to function as humanization and de-risking layers that refine hypothesis generation, prioritize candidate pathways, and inform model selection and endpoint definition [85]. Positioning animal studies within such a multi-tiered pipeline iteratively integrating in vivo models with human-relevant platforms offers a pragmatic strategy to reduce model-specific bias, strengthen mechanistic confidence, and accelerate translation of candidate therapies into clinically meaningful interventions.

## 9. Conclusions

Aortic aneurysm and dissection research has matured into a versatile toolkit of complementary models, each capturing different facets of disease biology. The next step is to move beyond descriptive pathology toward strategic integration, selecting models based on the research question, enforcing rigor and reproducibility, and bridging to human disease through biomarkers and validated endpoints. Embedding decision frameworks, reporting standards, and translational insights will be essential to elevate preclinical work into clinically actionable therapies. The overarching lesson is clear: there is no single “*best*” model, but rather the best model for the right question, at the right stage, and with the right endpoints. With the advent of precision genetic tools, multi-omics integration, and multicenter collaboration, the field is now positioned to leap from mechanistic discovery to transformative therapies that improve outcomes for patients with aortic diseases.

## Figures and Tables

**Table 1:** Decision grid linking scientific question to best-fit models.

Scientific Question	Best-Fit Models	Segment and Timeline	Rupture Frequency	Lipid Dependence	Typical Readouts	Key Caveats
**Mechanisms of rapid initiation**	AngII ± hyperlipidemia	Suprarenal; days–weeks	Suprarenal; days–weeks	Often yes	US growth, survival, histology	Not infrarenal; lipid sensitive
**ECM injury and** **biomechanics**	Elastase	Infrarenal; weeks	Infrarenal; weeks	No	Diameter, elastin/collagen, tensile tests	Surgical variability
**Rupture biology & strength failure**	Elastase + BAPN	Infrarenal; weeks–months	Infrarenal; weeks–months	No	Rupture/survival, ILT, biomechanics	Age/dose critical; supportive care
**Non-AT1** **endocrine drivers**	DOCA + NaCl_2_	Infra + thoracic; weeks	Infra + thoracic; weeks	No	Survival, MR signaling, oxidative stress	Electrolytes/BP confounder; age effects
**Dissection** **mechanisms**	BAPN (+ AngII)	Thoracic (desc. > asc.); days–weeks	Thoracic (desc. > asc.); days–weeks	No	False lumen, rupture, region-specific histology	Young mice; region heterogeneity
**Genetic HTAD** **pathways**	Fbn1/TGF-β/LRP1	Ascending/root; months	Ascending/root; months	No	Root dimension, histology, survival	Background- and stressor- dependent

## Data Availability

No new data were created or analyzed in this study.

## Abbreviations

The following abbreviations are used in this manuscript:

AAA	abdominal aortic aneurysm
TAA	thoracic aortic aneurysm
HTAD	heritable thoracic aortic disease
AngII	angiotensin II
BAPN	β-aminopropionitrile
DOCA	deoxycorticosterone acetate
ILT	intraluminal thrombus
GPVI	glycoprotein VI
